# Perturbation-based gait training to improve daily life gait stability in older adults at risk of falling: protocol for the REACT randomized controlled trial

**DOI:** 10.1186/s12877-020-01566-z

**Published:** 2020-05-07

**Authors:** Markus M. Rieger, Selma Papegaaij, Frans Steenbrink, Jaap H. van Dieën, Mirjam Pijnappels

**Affiliations:** 1 Department of Human Movement Sciences, Vrije Universiteit Amsterdam, Amsterdam Movement Sciences, Van der Boechorststraat 7, 1081 BT Amsterdam, The Netherlands; 2 Motek Medical BV, Hogehilweg 18C, 1101 CD Amsterdam, The Netherlands

**Keywords:** Treadmill, Exercise test, Walking, Accidental falls, Aging, Postural balance, Cognitive aging, Motor skills, Activities of daily living, Perturbation training

## Abstract

**Background:**

The European population is rapidly ageing. There is an urgent need for innovative solutions to reduce fall risk in older adults. Perturbation-based gait training is a promising new method to improve reactive balance responses. Whereas positive effects on task-specific dynamic balance recovery during gait have been shown in clinical or laboratory settings, translation of these effects to daily life gait function and fall risk is limited. We aim to evaluate the effect of a 4-week perturbation-based treadmill training on daily-life dynamic gait stability, assessed with inertial sensor data. Secondary outcomes are balance recovery performance, clinical balance and gait assessment scores, the amount of physical activity in daily life and falls incidence during 6 months follow-up.

**Methods:**

The study is a monocenter assessor-blinded randomized controlled trial. The target study sample consists of 70 older adults of 65 years and older, living in the community and with an elevated risk of falling. A block-randomization to avoid seasonal effects will be used to allocate the participants into two groups. The experimental group receives a 4-week, two times per week perturbation-based gait training programme on a treadmill, with simulated slips and trips, in combination with cognitive dual tasks. The control group receives a 4-week, two times per week treadmill training programme under cognitive dual-task conditions without perturbations. Participants will be assessed at baseline and after the 4-weeks intervention period on their daily-life gait stability by wearing an inertial sensor on the lower back for seven consecutive days. In addition, clinical balance and gait assessments as well as questionnaires on falls- and gait-efficacy will be taken. Daily life falls will be followed up over 6 months by a fall calendar.

**Discussion:**

Whereas perturbation-based training has shown positive effects in improving balance recovery strategies and in reducing laboratory falls, this study will contribute to investigate the translation of perturbation-based treadmill training effects in a clinical setting towards improving daily life gait stability and reducing fall risk and falls.

**Trial registration:**

NTR7703 / NL66322.028.18, Registered: January 8, 2019; Enrolment of the first participant April 8, 2019.

## Background

Falls are the leading cause of injuries in the growing population of older adults [1, 2]. Most falls occur while walking, caused by perturbations such as a trip [3]. Every year, one in three older adults aged over 65 years falls and this rate increases rapidly with age, leading to half of the people aged over 80 experiencing at least one fall per year [2, 4, 5]. In 2018, 108.000 older adults (65+) in the Netherlands suffered a severe injury associated with a fall and were treated in emergency departments or were admitted to hospitals [6]. Consequent health care costs are immense (€955 million in the Netherlands in 2018). It has been predicted that until 2050 the number of emergency care cases will increase up to 160.000 incidents per year [6]. Besides the risk of injuries and associated reductions in quality of life, an injurious fall may increase fear of falling [1], leading to a reduction in physical activity in daily life, which in turn could result in waning muscle strength and a further increased risk of falls [7].

To reduce fall incidence and rising health care costs it is imperative to prevent falls. Several studies have revealed that fall risk among older adults can be reduced by exercise interventions [8–11]. Conventional training programmes [12–14] have been shown to be effective in falls prevention, but require a relatively long and intensive training period and the positive effects after finishing the training period are hard to maintain [15–17].

A novel and promising approach in fall prevention is perturbation-based gait training [18]. Perturbation-based training challenges anticipatory as well as reactive balance control. Anticipatory balance control refers to adjusting posture and stepping behavior to overcome risky situations, i.e. changing to small and slow steps with a low center of mass position when walking over slippery floors, uneven surfaces or stairs. This might increase safety, but if a slip or trip does occur, one also needs to react adequately to recover balance. Reactive responses to unexpected perturbations during walking are task specific. It is known that task-specific training leads to strong learning effects [19]. Learning how to recover from a perturbation in daily life by exercising near-fall situations appears to improve motor responses [8]. Adaptions in reactive control and stability were found after a single session of perturbation training [20, 21]. Therefore, experiencing near-fall situations in a safe environment could facilitate the reactive control of balance in a daily life situation [22] and may have potential for fall prevention. Walking under dual-task conditions has been shown to improve both balance and cognitive performance [23] and is associated with better gait stability [24]. In a 4-week training study with older adults by Silsupadol et al. [25] the treadmill walking group with added dual task exercises significantly increased gait speed and balance scores on the Berg Balance Scale. In addition, there is evidence for the effectiveness of specific training to improve postural control in healthy older adults under dual-task conditions [26]. Adding a dual-task component to a motor-learning intervention can facilitate learning, automatization of the motor task and transfer to daily-life situations, by distracting participants from the primary motor-task [27, 28]. This has indeed been shown in 4- and 6-weeks conventional treadmill gait training programmes with dual-tasking, in which older adults improved in balance, gait and dual-task performance [25, 29].

Whereas most studies on perturbation training have shown positive effects on stability and gait performance in clinical or laboratory settings [30, 31], translation of these effects to daily life gait performance still remains unclear [31]. To obtain a comprehensive overview on whether and how perturbation-based gait training shows effects, we include measurements from the laboratory, clinical assessments on balance, gait, efficacy, falls and measurements from daily-life gait. To our knowledge this is the first RCT of its kind including older adults at risk of falling.

We aimed to design and test a 4-week treadmill training, with a combination of gait perturbation under dual task conditions, to maximize effects on balance and gait performance under real-life conditions.

A new treadmill module called REACT was developed for the C-mill (Motek Medical B.V., Amsterdam, the Netherlands), which allows belt perturbations in anterior-posterior direction in a safe manner. With this module, the C-Mill can accelerate or decelerate the belt quickly to mimic a slip or a trip and provoke a forward or backward balance loss, respectively. At the same time, cognitive dual tasks are provided to distract the participant and facilitate implicit learning, automatization and transfer to daily-life situations. Through a randomized clinical trial, we want to investigate the added value of a high number of perturbations in the experimental group under dual-task conditions compared to treadmill training under dual-task conditions without perturbations on daily life gait stability, balance performance, self-efficacy and falls.

The results of the randomized controlled trial proposed in this protocol may support application of perturbation-based treadmill training to enhance conventional (dual task) fall prevention training, facilitate automatization of skills and to reduce the training time needed to get a significant reduction of fall incidents. This may also reduce health care costs associated with falls.

## Methods

### Study design

The study design is a monocenter randomized control trial, assessor blinded, with two groups. It follows the recommendations of SPIRIT 2013. This trial will be executed at the physiotherapy practice” Motion Fysiotherapie & Preventie” in Uithoorn, The Netherlands.

### Participants

The target population consists of generally healthy older adults aged 65 and older, living in the community and identified at-risk of falling [32]. An English translation of the fall risk screening tool [32] can be found as a supplementary document (S1). Participants have no prior experience with perturbation training and will be excluded in case of (mild) cognitive impairment (< 24 points in the Montreal Cognitive Assessment) or any self-reported neurological, cardiovascular or pulmonary comorbidity (i.e. stroke, heart attack, hypertension) that occurred in the past 12 months, as well as orthopaedic complications (i.e. lower extremity fractures, joint replacements, low bone density) within the past 6 months. In- and exclusion criteria will be assed primarily through telephone screening. Participants will be asked for every criterion separately and also if they ever had received instructions from their general practitioner not to exercise or being physical active at medium intensity. See Table 1 for inclusion and exclusion details.

**Table 1:** Inclusion and exclusion criteria

Inclusion criteria	Exclusion criteria
Older adults aged ≥65 years Potential fall risk, as assessed by a short questionnaire [32]	Cognition < 24 points on the Montreal Cognitive Assessment (MoCA) Body mass over 135 kg Body height over 2.0 m Open skin lesion or bandage in the area of the harness contact Neurological comorbidities, e.g. Parkinson’s disease, multiple sclerosis, diabetic neuropathy, stroke, polyneuropathy Lower extremity fractures or torn ligaments in the past 6 months Not able to walk without walking aid at self-preferred speed Hip or knee joint replacement in the past 6 months Uncontrolled comorbid conditions, e.g. heart or lung/breathing diseases or low bone density, precluding physical activity at medium intensity

### Recruitment, randomization, blinding and treatment allocation

Potential participants will be invited by their physiotherapists to take part in this study. Additional recruitment through flyers at social gatherings and advertisement in local newspapers will be performed. Block-randomization will be used to avoid seasonal effects on the daily-life measurement. Seventy sealed envelopes in blocks of 10 (5 per group) will be prepared by a member of the research team, not involved in the recruitment, training or measurement. These sequentially numbered envelopes contain a random computer-generated unique identification number. A second matching envelope contains the group information. The blinded assessor will provide the second envelop to the trainer, so the group allocation is only revealed to the trainer, who opens the envelope at the first training day. Participants cannot be blinded to group allocation. If the group allocation of an individual participant will be revealed to one of the two trained blinded assessors due to unforeseen circumstances, the second blinded assessors will take over the post-intervention measurement.

### Informed consent

When showing interest to their therapist, participants will receive information via email and during a screening by telephone. If eligible with respect to the inclusion and exclusion criteria, participants will be asked to participate in the study and are willing to be randomized to one of the groups. All eligible participants will be given the time they need to consider participation and approach the investigator for questions. Patients who decide to participate sign the informed consent form and send it back to the investigator.

### Interventions

**Fig. 1:**
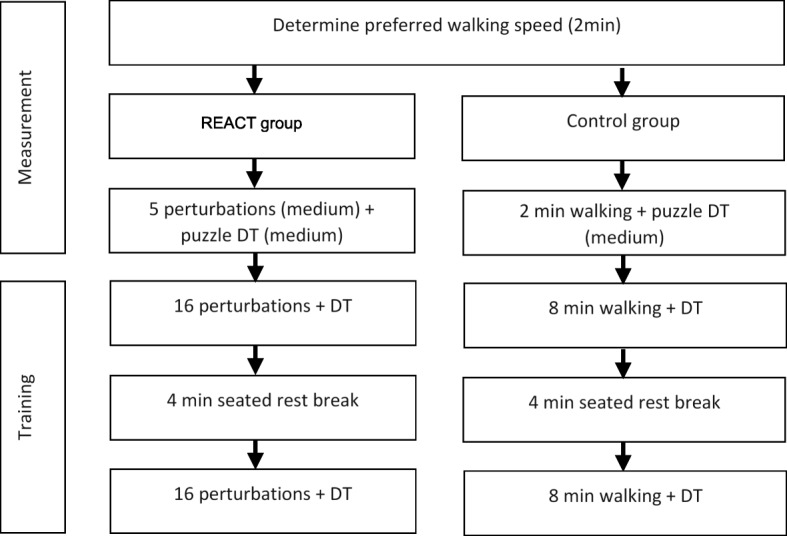
Overview over a training session in either the REACT or control group: DT = dual-task exercise; Measurements in the beginning of each session are performed with medium difficulty of the perturbations (REACT group) and of the cognitive dual-task exercise (both groups)

Specific trained physiotherapists will conduct the training. They will receive training on the use of the REACT module for the C-Mill and experience the REACT intervention themselves to be able to judge the challenge for a participant and adjust the difficulty of the training. Based on literature, a single session of perturbation training has been shown to reduce fall risk by 50% [10] and to have retention over 6 months [20]. Increasing the total training volume likely increases the effectiveness [33]. Therefore, participants in both groups will receive training twice a week for 4 weeks. Each training session will be split into two parts, a measurement and the training. The measurement will be done at the beginning of each session. First, the preferred walking speed will be determined and second, the gait of the REACT group will be perturbed with five perturbations at medium difficulty while playing the puzzle dual-task exercise on medium level (cf. section “Cognitive dual-task exercise” for description). The control group will walk for 2 min while playing the puzzle dual-task exercise on medium level. This will be done to track improvements over the training period and will take around 4 min. Please see Fig. 1 for an overview of one training session.

### Experimental intervention

The REACT group will receive a combination of perturbations and cognitive dual task training on the C-mill (Motek Medical BV, Amsterdam, The Netherlands). During these training sessions, their gait will be perturbed by quick accelerations and decelerations of the belt in anterior-posterior direction to mimic slips and trips. It has been suggested that introducing variation into exercise is more effective than repeating the same type of perturbation [8, 34]. Therefore, the REACT programme randomizes the perturbation types, while maintaining the same intensity. The random variations are: the type of perturbation (acceleration or deceleration), the time interval between perturbations (10–45 s), the perturbed leg (left or right), and the exact perturbation parameters (acceleration, duration). The intensity (10 difficulty levels of perturbations) of the training can be adjusted based on the participant’s performance at the discretion of the therapist. To distract the participant from the perturbations and to “automatize” the reactive responses, cognitive dual-tasks will be added (Fig. 4).

### Control intervention

The control group will receive a treadmill training under dual task conditions. This training will be provided with the same C-mill with REACT module, yet without any perturbations. Participants walk 2x 8 min on the treadmill twice a week for 4 weeks. Treadmill walking has been shown to improve patients gait function and balance [23–26, 29].

We ran a simulation of eight training sessions for 1000 participants, which resulted in an average duration for the five measurement perturbations of 2 min, an average duration for the training perturbations of 16 min (2x 8 min) and a total duration of a training session of 24 min (including 2 min warm up walking with adjusting comfortable walking speed and a 4-min rest break). The length of each part of the intervention can vary within one participant and between all participants, but on average it is negligibly small.

### Perturbations

**Fig. 2:**
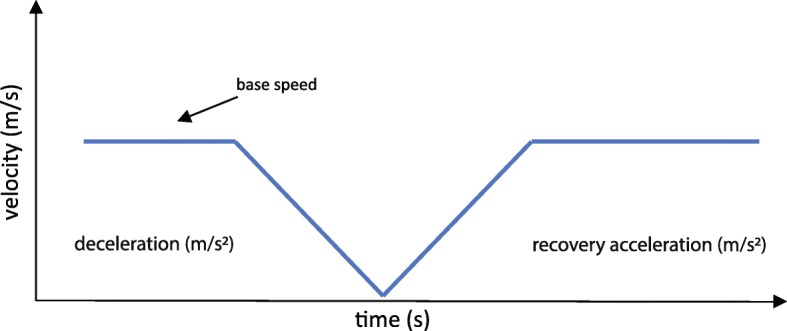
Illustrative drawing describing the velocity curve of a treadmill belt perturbation (deceleration inducing backward balance loss)

The belt perturbation will be randomly triggered on a left or right foot contact event during walking at a base speed. Contact will be detected by an algorithm utilizing the force plate embedded in the C-Mill. The perturbation profile is a linear acceleration of the belt (positive or negative) for a specified duration, followed by a return to the base speed with the same recovery acceleration. See Fig. 2 for an example. There are many different combinations of accelerations and durations, which result in perturbations of different difficulty levels.

**Fig. 3:**
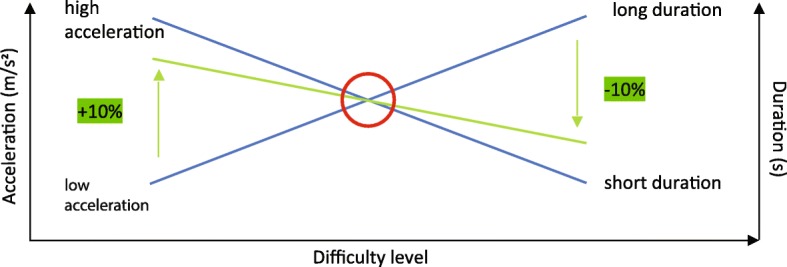
Figurative scheme of the variation within a perturbation difficulty level by changing the range values by 10%. A higher acceleration is combined a shorter duration and vice versa

Within a difficulty level, both decelerations and accelerations will be used. The difficulty level of the perturbations is mapped to an appropriate perturbation by varying the acceleration and duration of the perturbation using a fixed scheme (see Figs. 2 and 3). The difficulty level of a perturbation hence does not depend on walking speed.

The REACT can accelerate the belt with a maximum of ±6 m/s^2^. The perturbation duration can vary between 0.1–0.35 s. In a subjective test, perturbation settings (acceleration and duration) were identified for the highest [10] and lowest [1] difficulty level. Each difficulty level has a range for both parameters (acceleration, duration) to ensure small variations in the perturbations (see Table 2). Within this range, faster accelerations are combined with shorter durations and vice versa (see Fig. 3). In a pilot study we verified that the variation within one difficulty level did not result in a perceived difference in difficulty.

**Table 2:** Perturbation parameters for each difficulty level

Perturbation difficulty level	Acceleration in m/s^**2**^ (low)	Duration in seconds (low)	Acceleration in m/s^**2**^ (high)	Duration in seconds (high)
**1**	1	0.3	3	0.1
	-1	0.3	−2	0.15
**2**	1.5	0.31	3.33	0.13
	−1.5	0.31	−2.44	0.17
**3**	2	0.32	3.67	0.16
	−2	0.31	− 2.89	0.18
**4**	2.5	0.33	4	0.18
	−2.5	0.32	−3.33	0.20
**5**	3	0.34	4.33	0.21
	−3	0.32	−3.78	0.22
**6**	3.5	0.36	4.67	0.24
	−3.5	0.33	−4.22	0.23
**7**	4	0.37	5	0.27
	−4	0.33	−4.67	0.25
**e**	4.5	0.38	5.33	0.29
	−4.5	0.34	−5.11	0.27
**9**	5	0.39	5.67	0.32
	−5	0.34	−5.56	0.28
**10**	5.5	0.4	6	0.35
	−5.5	0.35	−6	0.3

Example:

For each difficulty level we defined minimum and maximum values for belt acceleration and duration. For example, at difficulty level 1, the acceleration can vary between 1 and 3 m/s^2^ and duration can vary between 0.3–0.1 s. A faster acceleration is combined with a shorter duration and vice versa. So, when the acceleration is 1 m/s^2^ the duration will be 0.3 s or with 3 m/s^2^ the duration will be 0.1 s. All variations in this range are possible as illustrated in Fig. 3.

### Cognitive dual-task exercise

**Fig. 4:**
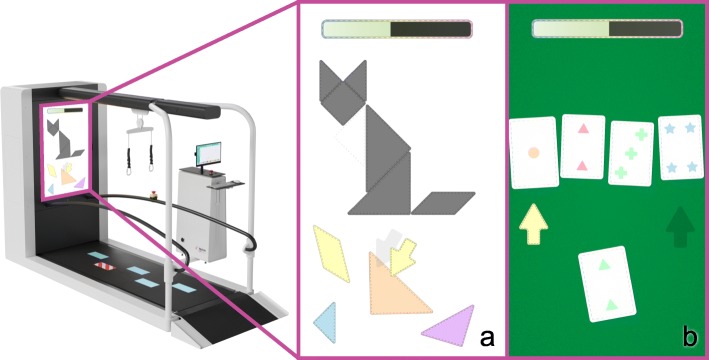
Illustration of the dual-task exercises presented on the front screen during both the REACT and the control intervention, a = ‘puzzle’, b = ‘card sorting’

Two cognitive dual-task exercises will be available to make training fun and challenging for the participant. The exercises will be controlled with a remote control with buttons to toggle between the answers and to select the correct answer. The aim of the ‘puzzle’ exercise (Fig. 4a) is to complete the presented puzzle by selecting the missing piece. The therapist can change the difficulty of the cognitive dual task to five different levels. When choosing higher difficulty levels, the visual appearance of the puzzle changes and more possible answers are presented that the participant has to choose from in less time. During the ‘card sorting’ exercise (Fig. 4b) the participant has to sort a presented card to one of four different piles of cards. These piles differ by the color, the shape and the number of shapes on the card. The aim is to figure out the correct rule to decide on which pile the card has to be placed. This can be either based on color, shape or number of shapes. The participant will get feedback on correctness of the matching. When a certain number of cards have been played, the rule changes randomly without notice. At higher difficulty levels the rule changes more often and the time to select an answer is reduced. The dual-task performance will be recorded quantifying the amount of correct answers and time to answer and will be used as co-variate in the analysis. Figure 4 shows an example of both dual task exercises.

### Training progress

Since training is most effective when the exercise is challenging (cf. overload principle), we aim to train participants with progressive challenges during the course of both training programmes [35]. The therapist will have several possibilities to challenge the participant during a training session and to increase the challenge over the training period. The participants start walking on their preferred walking speed that will be determined in the beginning of each training session. The therapist can encourage the patient to increase the speed from session to session to warrant progression in challenges. The ability to only walk at low speed is associated with fall risk [36], thus achieving a higher preferred walking speed over the training weeks is favored. All participants will be instructed to walk without use of the handrail, to maximize learning during the treadmill training [37].

Trainers will be instructed to keep the participant challenged over the training period by increasing either walking speed, dual-task difficulty or perturbation difficulty (only for experimental group). The instructions (in order of priority) are:

1. Try to train at a higher walking speeds every week. The participant may get used to treadmill walking and may tolerate higher walking speed over time. Try at least to reach over ground walking speed (from SPPB) at the end of 4 weeks. If possible, further increase is allowed.
2. The participant may tolerate stronger perturbation over time. Higher difficulty levels increase the intensity of the accelerations and decelerations. Try to increase difficulty every week. The patient should not need to grab the handrails to recover.
3. The dual task exercises are designed to distract the patient from the perturbations. By increasing the difficulty level, the patient’s focus shifts more to the dual task exercise and makes the perturbations more unpredictable. This increases the task specificity to daily life slips and trips. Try to increase this every week.

Trainers will monitor adherence throughout the programme and will encourage participants to reschedule a training session in case they missed one.

### Data collection and outcome measures

All participants undergo two measurements; one at entry of the study (baseline assessment, T1) and one after the 4-week intervention period (T2), within 7 days after the last training session. In addition, we will contact them after 6 months follow-up (T3). Table 3 highlights the measures collected and the time of assessment. To ensure data quality, two assessors, that will be blinded to group allocation, will be specific trained and perform all measurements. To evaluate the effect of the REACT perturbation-based treadmill training on daily-life dynamic gait stability, our primary outcome measure is a composite score of daily life dynamic gait quality, obtained from 1-week trunk accelerometry data [38]. This measure has been previously found to be an important predictor of future falls and allows for calculation of individual’s predicted survival fall rate [38]. Moreover, it has shown to have good reliability and potential as an outcome of daily life gait stability to evaluate intervention studies focusing on mobility, balance and falls [39]. Participants will receive an inertial sensor (Dynaport MoveMonitor, McRoberts BV, The Netherlands) at the baseline and post-intervention assessments. They will be instructed to wear the sensor at their lower back with an elastic band around the waist for seven consecutive days, preferably day and night, except during water activities and return the MoveMonitor by mail. The MoveMonitor registers trunk accelerations in vertical (VT), mediolateral (ML) and anteroposterior (AP) directions, with a sample frequency of 100 samples/s and range of +/− 6 g. Episodes of locomotion are identified using the manufacturers algorithm that was previously validated [40]. Only the locomotion episodes that last 10 s or longer will be selected and divided into epochs of 10 s and then gait quality characteristics will be calculated for each of these 10-s epochs. Subsequently, gait quality characteristics will be estimated as median values over the week and the gait quality composite score will be calculated, based on a weighted sum of autocorrelation at stride frequency, power at step frequency, root mean square of the accelerations and index of harmonicity [38, 39].

**Table 3:** Patient flow and overview of outcome measurements and time of assessment

	T0	T1	TM	T2	T3
**Recruitment**	**X**				
**Assessment in/exclusion & population conditions** Participant’s characteristics (age, gender, weight, height, 12 month fall history), MoCA	**X**				
**Sign informed consent form**	**X**				
**Balance assessment**: SPPB, Mini-BEST, FSST		**X**		**X**	
**Questionnaires** FES-I, GSE, mGES, KAP		**X**		**X**	
**Randomization**		**X**			
**Training assessment** Treadmill parameters Dual-task performance Recovery performance			**X**		
**Questionnaires** Satisfaction about the training				**X**	
Falls calendar (6-months follow up period)					**X**
**Measurement of daily life gait quality and physical activity** Inertial sensor (MoveMonitor) for 7 consecutive days		**X**		**X**	

*T0* prior to study, *T1* baseline (pre-intervention), *TM* Training Measurements, for each of the 8 sessions during the 4-week training period, *T2* post-intervention, *T3* follow-up (6-months post-intervention)

Secondary outcomes will include the amount of daily physical activity that can be derived from the accelerometry data from the same daily-life measurement at baseline and post-intervention [41, 42]. This measure contains the wear time of the MoveMonitor, the time being active (i.e. standing and shuffling), in locomotion (i.e. walking, stair walking, cycling) and time spent in sedentary behavior (i.e. sitting and lying).

Balance and gait will be assessed at baseline and post-intervention with three clinical tests:

1. The Mini Balance Evaluation System Test (MiniBESTest) is a 14 item test that aims to target and identify six different balance control systems. It includes four subscales: transitions/anticipatory postural control, reactive postural control, sensory orientation and stability in gait [43, 44].
2. The Short Physical Performance Battery (SPPB) is a tool to measures gait speed, chair rise and balance. The SPPB has been shown to have predictive validity showing a gradient of risk for mortality, nursing home admission, and disability [45, 46]
3. The Four Square Step Test (FSST) a reliable, valid, easy to score and quick to administer test for ones standing balance, mobility and falls risk, that requires little space and needs no special equipment. It contains stepping over low objects (2.5 cm) and movement in 4 directions [47–49]

During both assessments, fear of falling will be measured with the Falls Efficacy Scale-International (FES-I) [50] and the Dutch version of the modified gait efficacy scale (mGES) [51]. These questionnaires measure the level of concern about falling during social and physical activities of daily living. The general self-efficacy scale (GSE) [52] will be used to measure self-efficacy in daily-life situations, and the Keele assessment (KAP) [53] to measure participation.

During the training period, in each session of the REACT group, balance recovery will be assessed for each of the perturbations by the quantified recovery performance (QRP) using a single one-directional force plate embedded in the C-Mill. Continuous COP position data in anterior-posterior direction will be first smoothed with a second-order 6 Hz low-pass Butterworth filter and then filtered with a second-order 0.5 Hz high-pass Butterworth filter to remove the variability of the position of the subject on the treadmill. A template will be created of the average COP trajectory over five seconds of unperturbed walking. The COP trajectory of the first five seconds after the perturbation will be aligned with this template using cross-correlation. The maximum in the cross-correlation function represents the QRP. A higher correlation of the two COP trajectories indicates a better recovery from the perturbation back to one’s normal gait pattern.

Finally, daily life fall incidences will be followed up for 6 months after the last training session. At the post-intervention assessment, participants receive a calendar in which they will be instructed for the following 6 months to state when a fall occurred, in which situation, in which direction, if the fall was due to slipping, tripping or feeling dizzy and if it resulted in an injury. Participants will be contacted by phone at the end of the 6 months follow-up period to ask if there were problems filling in the falls calendar and remind them to return it by mail.

### Data management

Every participant will receive a computer-generated unique identification code at the baseline assessment. All data will be collected anonymously with this identification code. For each identification code a logbook will be started in which test results from baseline and post-intervention assessments will be collected as well as the documentation on each training session. In terms of data quality, a trained project-assistant will verify the data entries and check the digitalized version after all trainings and assessments are finished. If data are missing, the assistant will check the logbook. For data cleaning, the investigator is blinded to group allocation. Participants return the MoveMonitor after the measurement is finished. Inertial sensor data will be uploaded to the McRoberts’ server for processing of activity classification of physical activity (walking, climbing stairs, standing up, shuffling, cycling) and sedentary behavior (lying, sitting). We will download the classification from their server and save it together with the raw data on a password protected external hard disk. Data that will be generated during the training will be automatically saved on the C-Mill and a backup on a password protected external hard disk will be done on a regular basis.

### Sample size

To compare the experimental and control intervention on fall risk (obtained from daily-life dynamic gait quality), we assume effect sizes based on evidence from previous interventions. For conventional training, a 29% reduction in fall risk is the pooled estimate based on a review by Gillespie and co-workers [9]. For the experimental group, the effect size based on a previous perturbation study by Pai and colleagues [10] is estimated at a 50% reduction of falls, which implies survival fall rate changes from 74 to 87%. We will use the composite score of daily life dynamic gait quality [38, 39], as the primary outcome variable. This composite score has been shown to predict individual’s survival fall rate. Hence, expected effects on fall risk (1-survival fall rate) can be related to expected changes in the composite score. We will use a 2-way repeated measures ANOVA with training (control group vs experimental group) as between subject factor and time-point (pre- vs post-training) as within factor on these composite scores. Given the difference in effects of the training interventions considered above, a medium effect size (f = 0.25) for the interaction of intervention and time-point is expected. However, our perturbations are less strong than described in the study by Pai et al. [10], therefore we use a slightly smaller effect size (f = 0.21) in the power analysis.

Assuming α = 0.05, β = 0.95 and using a conservative estimate for the correlation between time points of 0.6, based on van Schooten et al. [38], G*Power [54], version 3.1.9.2, indicates a total sample size of 62 (31 per group). To account for dropouts, a total number of 70 participants will be included.

### Statistical analysis

Data will be analyzed using SPSS (SPSS Inc., Chicago, IL, USA) and custom MATLAB (version R2018a; MathWorks Inc., Natick, MA, USA) scripts. The data analysis is designed to determine the effects of the REACT perturbation-based gait training programme and the conventional treadmill training on dynamic gait stability and physical activity in daily life and on the performance in clinical gait and balance parameters. The analysis will be based on on-treatment data of complete data sets of people who fulfilled the protocol. This type of analysis allowed us to evaluate the effects with optimal adherence, as participants are encouraged to reschedule a training session in case they miss one. We therefore expect low risk of bias due to minimal drop-out. Number and reason for drop-out and for incomplete data-sets on primary outcome will be provided.

The focus of the analysis will be on the composite score of daily-life gait quality. We will provide an indication of the reduction in fall risk related to improvements in the primary and secondary outcome parameters. For missing values on secondary outcomes, we will use average values or linear regression analyses.

Data at baseline and post-intervention will be analyzed using descriptive statistics such as mean (or median), standard deviation (or interquartile range) and percentage to describe the characteristics of the participants and their performance on assessments. Comparisons within and between groups at baseline and post-intervention will be done using ANOVAs.

Effects of training on the primary outcome, the composite score of daily-life gait quality derived from one-week of inertial sensor data, will be tested using a 2-way mixed-design ANOVA with baseline vs post-intervention as within-subject factor and group as a between-subject factor.

For secondary outcomes, results of baseline and post-intervention measurements will be compared with a 2-way mixed ANOVA as above. For the outcome of falls incidence during follow-up, the number of falls will be analyzed using negative binomial regression to estimate the difference in falls rates between the two groups. Proportion of fallers between groups will be compared using the incidence rate ratio statistic.

### Adverse events

Adverse events are defined as any undesirable experience occurring to a subject during the study, whether or not considered related to the experimental intervention. All adverse events reported spontaneously by the subject or observed by the investigator or his staff will be recorded.

### Data monitoring committee

Based on the non-critical indication (low risk intervention), the participant populations (relatively healthy older adults without any diagnosed comorbidities or cognitive impairments), and relatively short duration of the trial (4 week intervention with follow-up over a total duration of 6 months), a data monitoring committee (DMC) was not required.

### Trial status

Enrolment into the study started on April 8, 2019. We completed the recruitment, training and T1 and T2 data collection by November 18, 2019, with a follow-up period on falls incidence (T3) until the end of May 2020.

## Discussion

### Strengths

This study assesses an innovative perturbation-based gait training in clinical practice in a physiotherapy setting. It follows a strict RCT design with blinding where possible. The training follows the key factors for successful motor learning [55]; the training is task specific (mimics real life slips and trips in a safe environment), has a high training intensity (difficulty is adjusted to the participant’s performance), is variable (perturbation direction and magnitude are randomized), and provides immediate feedback for the therapist on a participant’s recovery performance to set the difficulty level. In our study, the participant is not informed on the recovery performance during the training. The added cognitive dual-task may help to automatize the reactive balance recovery strategies. While single session interventions already yielded significant improvements in balance and reduction in laboratory falls, we choose to have eight training sessions to be able to identify a possible plateau in direct training outcomes. The training is designed to take less than 30 min. This supports future clinical application as it can be easily performed within a normal therapy session. Besides evaluating task-specific effects of perturbation training, our main outcome measure focusses on the ability to transfer these effects to daily life gait performance, by using ambulatory measurements of dynamic gait stability and physical activity. In addition, the 6 months follow up will give us some insight into the falls reduction in daily life, although the study will lack statistical power for this outcome.

### Weaknesses

Even though trainers will be trained intensively to correctly judge the training challenge for participants, there will be variance in the training progression due to individual judgement. Recommendations on how to adapt training challenge will be provided, but no strict guidelines. Although this may induce variance, it also allows individualized training and enhances acceptability of the training protocol in clinical practice by therapists. Disadvantages of the training are that it is device-dependent and group training is not possible. This setup is limited in perturbing in different directions, but as we stated in the introduction the requirements in clinical practice are a small and affordable device. More advanced systems with more options to perturb the gait in different directions do not fulfill these requirements as they are much bigger, complex in controlling and entail high costs. However, the C-Mill used in this trial would be able to provide standing perturbations also in medio-lateral direction after a software update to be able to train reactive balance responses in the medio-lateral direction. Potential sources of bias include participant selection and loss to dropout and during follow-up.

## Supplementary information

**Additional file 1.**

## Data Availability

Only the investigator and study coordinator will have access to the anonymized final full trial dataset. Sharing of anonymized questionnaires is planned within one partner of the EU project “KeepControl” respecting participant confidentiality. A minimal dataset that allows to interpret, replicate and build upon the findings reported in the article are available from the corresponding author on reasonable request.

## Abbreviations

Mini-BESTest: Mini balance evaluation system test
SPPB: Short physical performance battery
FSST: Four square step test
FES-I: Falls Efficacy Scale International
mGES: Modified gait-efficacy scale
GSE: General self-efficacy scale
KAP: Keele assessment of participation
MoCA: Montreal cognitive assessment
DT: Dual-task
VT: Vertical
ML: Medio-lateral
AP: Anterior-posterior
QRP: Quantified recovery performance
COP: Center of pressure
ANOVA: Analysis of variance
RCT: Randomized controlled trial
NWO: Dutch Organisation for Scientific Research
EU: European Union
