# Supplementary material for: Perturbation-based gait training to improve daily life gait stability in older adults at risk of falling: protocol for the REACT randomized controlled trial
Source: BMC Geriatr. 2020 May 7;20:167. doi: 10.1186/s12877-020-01566-z (PMC7203817; doi:10.1186/s12877-020-01566-z)

Fall Risk Assessment

The fall risk assessment takes less than a minute and is a quick way to evaluate if the client has an increased fall risk. If this test shows that there is and increased risk of falling you can perform the fall analysis to be able to give specific advice.

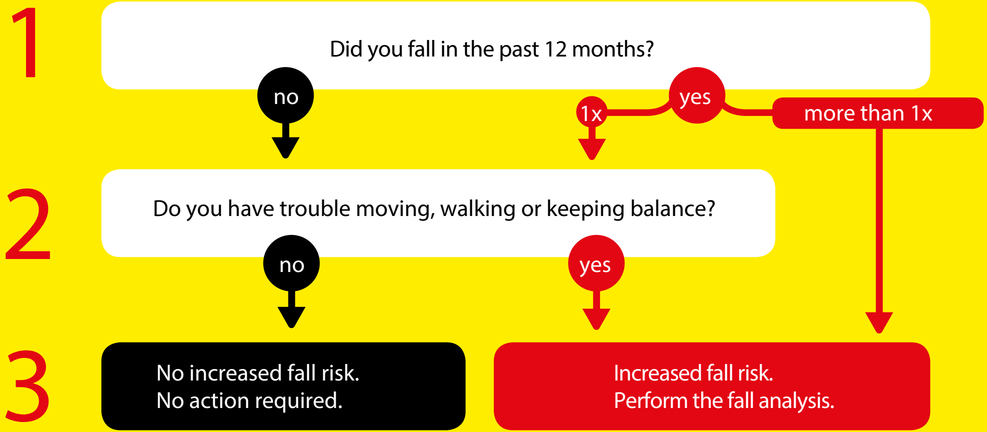

Supplement: Supplementary file 1 — Additional file 1. [file 12877_2020_1566_MOESM1_ESM.pdf]
